# Revealing the Role of Vapor Flux in Chemical Vapor Deposition Growth of Bi_2_O_2_Se for Photodetectors

**DOI:** 10.3390/nano15080567

**Published:** 2025-04-08

**Authors:** Qin Huang, Jiqing Nie, Jian Li, Meng Wang, Changyuan Ding, Haiyan Nan, Xiaofeng Gu, Zhengyang Cai

**Affiliations:** School of Integrated Circuits, Jiangnan University, Wuxi 214122, China; 6231916004@stu.jiangnan.edu.cn (Q.H.); 6231916043@stu.jiangnan.edu.cn (J.N.); 6231916034@stu.jiangnan.edu.cn (J.L.); 6221916039@stu.jiangnan.edu.cn (M.W.); dcy20002022@163.com (C.D.); jnanhaiyan@jiangnan.edu.cn (H.N.); xgu@jiangnan.edu.cn (X.G.)

**Keywords:** two-dimensional materials, chemical vapor deposition, Bi_2_O_2_Se, vapor reaction, photodetectors, density functional theory

## Abstract

Two-dimensional (2D) materials are regarded as key foundational materials for next-generation optoelectronic devices. As a promising new type of 2D layered semiconductor, Bi_2_O_2_Se has emerged as a strong candidate for high-performance opto-electronic devices due to its high carrier mobility, tunable bandgap, and excellent environmental stability. However, achieving precise control over Bi_2_O_2_Se growth to obtain high-quality Bi_2_O_2_Se remains a challenge in the field. In this study, we employed chemical vapor deposition (CVD) to grow thin-layer 2D Bi_2_O_2_Se flakes. We further used a transport model and thermodynamic Arrhenius fitting to analyze the relationship between vapor flux and the properties of the flakes. Density functional theory was used to study the electronic structure of the as-grown samples. The electrical and optoelectronic results demonstrate that Bi_2_O_2_Se-based FETs exhibit good performance in terms of mobility (129 cm^2^V^−1^s^−1^), on/off ratio (4.51 × 10^5^), and photoresponsivity (94.98 AW^−1^). This work provides a new way to study the influence of vapor flux on the sizes and shapes of Bi_2_O_2_Se flakes for photodetectors.

## 1. Introduction

Two-dimensional (2D) materials have attracted significant attention due to their unique properties, including layer-dependent electronic structures, tunable photonic properties, electrocatalytic activity, and mechanical flexibility [1,2,3]. These features have enabled advancements in electronic and optoelectronic devices [4,5,6] as well as sensors [7,8]. Graphene, renowned for its high carrier mobility [9], has limitations in field-effect transistors (FETs) due to its zero bandgap, which hampers effective on/off switching. Black phosphorus, with a thickness-dependent bandgap of 0.3–2 eV and high on/off ratios, offers better potential but suffers from air instability and challenges in large-scale film growth [10,11,12]. Transition metal dichalcogenides (TMDCs) have shown promise in optoelectronics due to their favorable properties but are constrained by relatively low carrier mobility [13,14]. These limitations highlight the need to explore novel 2D materials with tunable bandgaps, high carrier mobility, and environmental stability, which are essential for next-generation optoelectronics.

In recent years, Bi_2_O_2_Se has gained attention in optoelectronics due to its high carrier mobility [15] (up to 28,900 cm^2^V^−1^s^−1^ at 2 K), moderate bandgap [16] (~0.8 eV), and excellent environmental stability [15,17,18]. These properties make Bi_2_O_2_Se a promising candidate for high-performance optoelectronic devices, though research on these applications remains limited compared to materials like MoS_2_ and WSe_2_. Initial studies by Oppermann et al. involved synthesizing bulk Bi_2_O_2_Se via hot-pressing and analyzing its semiconductor properties and phase diagram [19]. Subsequent work explored its potential in transport and thermoelectric applications [20,21]. Recently, advancements in chemical vapor deposition (CVD) have enabled the growth of 2D Bi_2_O_2_Se [22,23,24], revealing its high carrier mobility and low electron effective mass. The above achievements highlight the need for further research to fully realize Bi_2_O_2_Se’s potential in high-performance optoelectronics.

The performance of Bi_2_O_2_Se is strongly influenced by the preparation method. Wet chemical synthesis stands out for its low cost and high yield, making it an attractive option for large-scale production. However, it faces challenges in achieving uniform thickness and size across samples [25,26]. Physical vapor deposition (PVD) has been used for the heteroepitaxial growth of Bi_2_O_2_Se thin films [27], but its slow growth rates and high production costs limit its practicality. In contrast, CVD offers advantages in terms of controllability and scalability. It enables the precise regulation of critical parameters, such as thickness, size, and uniformity. Studies have demonstrated that optimizing CVD growth parameters, such as reaction temperature, precursor concentration, and pressure, can grow Bi_2_O_2_Se flakes with high crystallinity, uniform thickness, and tunable properties. For example, Wu et al. synthesized ultra-high-mobility Bi_2_O_2_Se using a controlled CVD process, achieving effective control over thickness, nucleation sites, and size by adjusting growth parameters [22]. Similarly, Khan et al. developed a salt-assisted CVD, successfully growing atomically thin Bi_2_O_2_Se materials [28]. They further employed a catalyst-free CVD method to grow nearly monolayer Bi_2_O_2_Se nanoribbons, demonstrating the ability to precisely control the morphology of Bi_2_O_2_Se [23]. These advancements highlight the versatility and potential of CVD as a reliable method for growing high-quality Bi_2_O_2_Se with tailored properties, paving the way for its integration into advanced optoelectronics.

In this paper, we develop an atmospheric pressure CVD method for the growth of 2D Bi_2_O_2_Se flakes. By controlling key parameters such as the growth temperature and gas flow rate in the furnace, the growth of Bi_2_O_2_Se can be precisely regulated with varying shapes and thicknesses. To investigate the underlying mechanisms of how the parameters affect the properties of as-grown Bi_2_O_2_Se, we used thermodynamic and transport models relating the growth temperature and gas flow rate to the sizes and shapes of as-grown Bi_2_O_2_Se flakes. The Bi_2_O_2_Se-based FETs exhibited a high on/off ratio of approximately 4.51 × 10^5^ and a high carrier mobility of 129 cm^2^V^−1^s^−1^. Furthermore, the photodetectors made of Bi_2_O_2_Se achieved a maximum responsivity of 94.98 AW^−1^ and a maximum detectivity of 1.46 × 10^9^ Jones. The abovementioned excellent device performance of the Bi_2_O_2_Se flakes indicates their potential for applications in the fields of electronics and optoelectronics.

**Figure 1:**
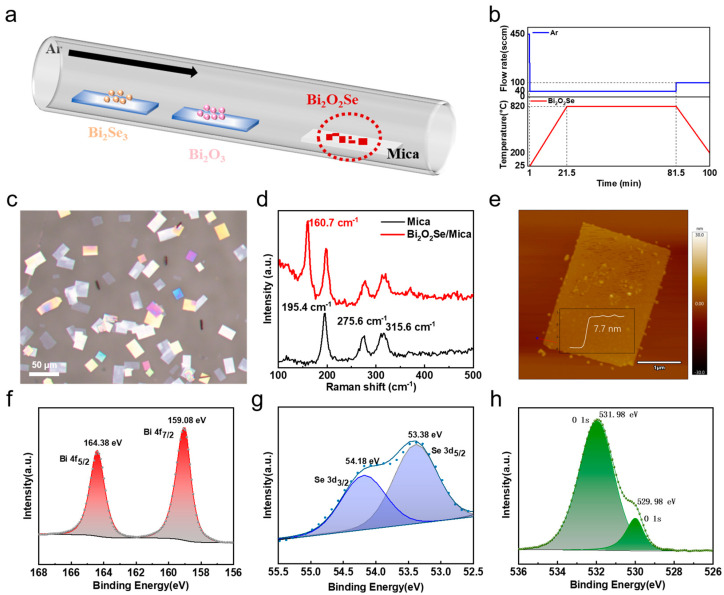
Growth and characterization of layered Bi_2_O_2_Se flakes. (**a**) Schematic of CVD growth of Bi_2_O_2_Se flakes. (**b**) Growth parameters regarding temperature and gas flow rate during CVD process. (**c**) Representative OM image of Bi_2_O_2_Se flakes grown on mica substrate. (**d**) Raman spectra of Bi_2_O_2_Se flakes and pristine mica substrate under 532 nm laser excitation. (**e**) Typical AFM image of Bi_2_O_2_Se flakes with thickness of 7.7 nm. (**f**–**h**) XPS spectra of thin-layer Bi_2_O_2_Se flakes.

Figure 1a shows the experimental setup for the growth of Bi_2_O_2_Se flakes. The electrostatic interaction between mica and Bi_2_O_2_Se facilitates the lateral growth of Bi_2_O_2_Se flakes [22,29,30]. Therefore, freshly cleaved mica was chosen as a suitable substrate for the growth of atomically thin 2D Bi_2_O_2_Se (details are provided in Section 2). Dual pre-cursors, Bi_2_O_3_ and Bi_2_Se_3_, were used as the co-evaporation sources for Bi_2_O_2_Se growth, and the mica substrate was placed downstream for deposition. During growth, the temperature and gas flow rate inside the furnace were accurately monitored, with their temporal variations shown in Figure 1b. A typical growth temperature was 820 °C, with a gas flow rate of 40 sccm, and the growth time was maintained for 1 h, which allowed for the growth of high-quality Bi_2_O_2_Se flakes. Figure 1c shows a representative optical microscope (OM) image of Bi_2_O_2_Se flakes, which have a square or rectangular shape, with an average domain size of approximately 30 µm. As shown in Figure S1 (Supporting Information), the largest domain size of the Bi_2_O_2_Se flakes exceeded 220 µm. The Raman spectra in Figure 1d show a characteristic peak centered at 160.7 cm^−1^, which is attributed to the A_1_g vibration mode of Bi_2_O_2_Se, with the reference being the pristine mica substrate. Raman spectra of Bi_2_O_2_Se with different flakes are shown in Figure S2a,b (Supporting Information), showing characteristic peaks at 158.1 cm^−1^ and 160.0 cm^−1^, respectively. The variations in the peak can be ascribed to the effects of Bi_2_O_2_Se thickness. An atomic force microscopy (AFM) image shows that the thickness of Bi_2_O_2_Se flakes is approximately 7.7 nm (Figure 1e). Considering the thickness of a Bi_2_O_2_Se unit is 0.61 nm [15,31], 7.7 nm corresponds to about 13 layers. The thinnest Bi_2_O_2_Se flakes obtained in our growth were 4 nm, or about 6–7 layers, as shown in Figure S3 (Supporting Information). To determine the chemical state of the as-grown 2D Bi_2_O_2_Se flakes, X-ray photoelectron spectroscopy (XPS) was conducted. As shown in Figure 1f–h, peaks corresponding to Bi 4f_5/2_ and Bi 4f_7/2_ are centered at 164.38 eV and 159.08 eV, respectively, consistent with the Bi element in bismuth oxide [32]. Also, the O 1s orbit shows peaks at 531.98 eV and 529.98 eV, and the Se 3d orbit shows peaks at 54.18 eV and 53.38 eV. These binding energies of Bi, O, and Se elements are consistent with those in Bi_2_O_2_Se [33]. The above results confirm the successful growth of Bi_2_O_2_Se flakes on mica substrates.

**Figure 2:**
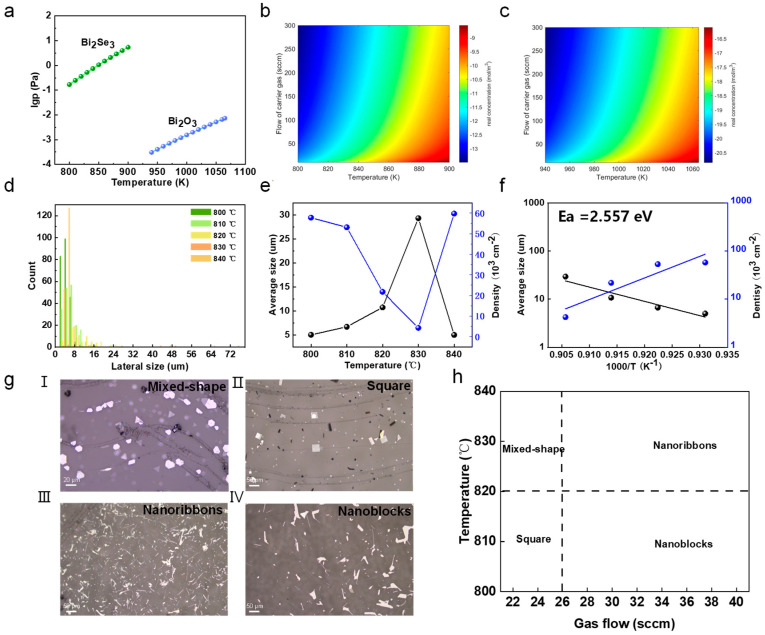
The controlled growth of Bi_2_O_2_Se flakes. (**a**) The statured vapor pressure of Bi_2_Se_3_ and Bi_2_O_3_ precursors. (**b**,**c**) The actual vapor concentration of (**b**) Bi_2_Se_3_ and (**c**) Bi_2_O_3_ precursors at given temperatures and gas flow rates. (**d**) The statistics of the Bi_2_O_2_Se domain size under varied growth temperatures ranging from 800 °C to 840 °C. (**e**) The average size and nucleation density of Bi_2_O_2_Se flakes grown under varied temperatures. (**f**) Arrhenius plots of average domain size versus 1000/T under varied growth temperatures. (**g**) Typical optical images of Bi_2_O_2_Se flakes with different shapes, namely mixed-shape, square, nanoribbons, and nanoblocks and (**h**) corresponding growth parameters like temperature and gas flow rate.

Understanding the vapor concentration of precursors is crucial in controlling the CVD growth of 2D Bi_2_O_2_Se. We initially plotted the saturated vapor pressure versus temperature for both Bi_2_Se_3_ and Bi_2_O_3_ precursors, as shown in Figure 2a [34]. Then, we used it to calculate the actual vapor concentration based on a transport model based on Equation (1) [35].

(1) $$C_{i}=C_{i}^{*}[1-\exp\left(-\frac{K_{i}}{\bar{u}}\frac{A_{h}}{A}\right)]$$

where $C_{i}^{*}$ represents the saturated vapor concentration, $\bar{u}$ refers to the average flow velocity of the carrier gas, and *K_i_* stands for the mass transfer coefficient. *A_h_* represents the area of the precursor (assumed to be 1 cm × 1 cm). A represents the cross-sectional area at the site where the precursor is located. As exhibited in Figure 2b,c, we can observe that the vapor phase concentration is in the range of 10^−13^–10^−9^ mol/m^3^ for Bi_2_Se_3_ and 10^−20^–10^−16^ mol/m^3^ for Bi_2_O_3_ precursors, and this concentration increases with the temperature’s increase and gas flow rate’s decrease. Hence, the reaction concentration can be precisely controlled by modulating the growth parameters of temperature and gas flow rate. First, we investigated the effects of growth temperature. Figure 2d shows the lateral size distribution of Bi_2_O_2_Se flakes grown at temperatures ranging from 800 to 840 °C. The corresponding OM and individual distribution are given in Figure S4 (Supporting Information). Most Bi_2_O_2_Se flakes were relatively small in size. Specifically, Figure 2e shows the average size and nucleation density of the Bi_2_O_2_Se flakes versus the growth temperature. The increase in temperatures results in larger flakes with a lower nucleation density, while lower temperatures yield smaller flakes with higher nucleation density. To gain further insights into the growth mechanism of Bi_2_O_2_Se, we conducted a thermodynamic Arrhenius analysis of the average domain size versus 1000/T, as shown in Figure 2f [36]. The reaction barrier for growth was extracted to be 2.557 eV by fitting the formula in Equation (2):

(2) $$\log(D/D_{0})=C-(\frac{E_{a}^{exp}}{1000k_{B}\ln10})(\frac{1000}{T})$$

where *D* is the average size of Bi_2_O_2_Se flakes, *T* is the growth temperature, *D*_0_ = 1 μm is used for normalization, *C* is a constant, and $E_{a}^{exp}$ denotes the activation energy. *k_B_* represents the Boltzmann constant. The above results indicate that the growth is a thermally activated growth mechanism. We also studied the effects of both temperature and gas flow rate on the shapes of the as-grown Bi_2_O_2_Se flakes. Figure 2g shows the OM images of Bi_2_O_2_Se flakes with different shapes, such as mixed-shape, square, nanoribbons, and nanoblocks. These variations in shape are a result of the interplay between the temperature, gas flow rates, and vapor concentration during the CVD process. As shown in Figure 2h, higher temperatures and slower gas flow rates tend to favor the growth of square and nanoblock flakes, while mixed-shape flakes are typically observed at high temperatures and gas flow rates. Nanoribbons are typically observed at lower temperatures with very high gas flow rates. The above results reveal the role of vapor flux in controlling the sizes and shapes of as-grown Bi_2_O_2_Se flakes.

**Figure 3:**
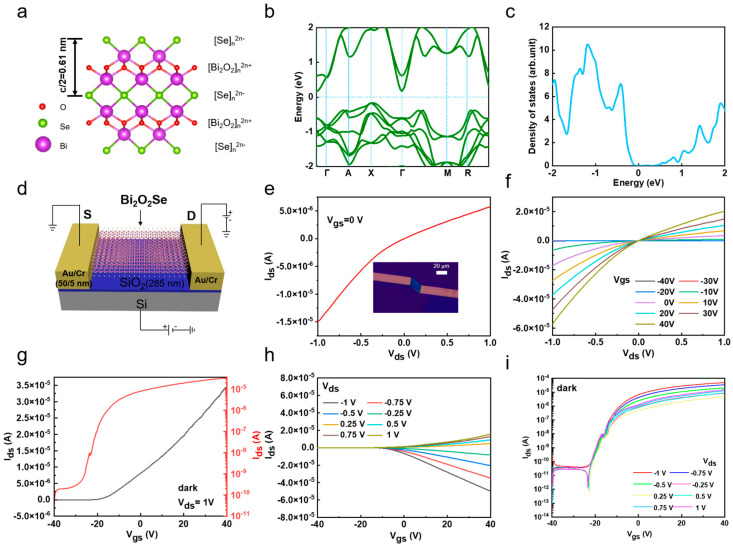
Electrical performance of 2D Bi_2_O_2_Se photodetectors. (**a**) Schematic of layered Bi_2_O_2_Se crystal structure. (**b**,**c**) Band structure and density of states of Bi_2_O_2_Se. (**d**) Schematic of back-gate Bi_2_O_2_Se flake-based FET. (**e**) Output characteristics of device (Ids-Vds) at zero gate voltage, while inset OM image shows channel length and channel width of Bi_2_O_2_Se flake-based FET device, which are 18.28 µm and 12 µm, respectively. (**f**) Output characteristics of FET device with Vgs ranging from −40 V to 40 V. (**g**) Transfer characteristics of device (Ids-Vgs) at Vds = 1 V, displayed in both linear and logarithmic scales. (**h**,**i**) Ids-Vgs family curves of device as function of Vds, shown in linear and logarithmic scales.

Based on the above CVD-grown Bi_2_O_2_Se flakes, we studied their electrical properties by fabricating Bi_2_O_2_Se-based FETs. Bi_2_O_2_Se is a layered material composed of alternating [Bi_2_O_2_]n^2n^⁺ and [Se]n^2n−^ layers stacked along the c-axis, which form covalent bonds through electrostatic interactions, as visualized in Figure 3a. Previous studies have extensively employed density functional theory (DFT) to calculate the band structure of 2D semiconductors [37,38]. Hence, we used density functional theory (DFT) to calculate the band structure and density of states of Bi_2_O_2_Se, revealing that it has an indirect bandgap of approximately 0.34 eV, with the valence band maximum at the X point and the conduction band minimum at the Γ point (Figure 3b,c). It should be noted that the bandgap of bulk Bi_2_O_2_Se was measured to be ~0.85 ± 0.05 eV using scanning tunneling spectroscopy (STS) [17]. We then fabricate the back-gated device by transferring Bi_2_O_2_Se flakes from mica to a 300 nm thick SiO_2_/Si substrate. Figure 3d shows a schematic of Bi_2_O_2_Se flake-based FETs. Figure 3e shows the output characteristics (Ids-Vds) of the Bi_2_O_2_Se device measured at room temperature under zero gate bias, with a current reaching up to approximately 5.75 × 10^−6^ A at a bias of 1 V, indicating excellent electrical performance. The asymmetry of this curve demonstrates a Schottky barrier existing at the electrode–channel contact interface. The inset of Figure 3e shows an OM image of a Bi_2_O_2_Se FET device with a channel length of 18.28 µm and a width of 12 µm. The effective area is 219.36 µm^2^. When the back-gate voltage was adjusted from −40 V to 40 V in a 10 V step, it was observed that the drain current (Ids) strongly depended on Vgs, demonstrating its back-gate controllability (Figure 3f). The nonlinear features of these Ids-Vds curves further suggests the presence of a Schottky barrier, which may be due to the electrode’s oxidation during the deposition process. Figure 3g shows the transfer characteristics (Ids-Vgs) at a bias voltage Vds of 1 V, displayed in both linear and logarithmic scales. The n-type behavior of the Bi_2_O_2_Se device causes Ids to increase (decrease) with a positive (negative) Vgs. The on/off ratio at a bias of 1 V is approximately 4.51 × 10^5^, and the field-effect mobility (µ) extracted from this curve is 129 cm^2^V^−1^s^−1^. Furthermore, Figure 3h,i show the Ids-Vgs curves at various bias voltages Vds ranging from −1 V to 1 V with a step of 0.25 V in both linear and logarithmic scales, further confirming the n-type characteristics of the Bi_2_O_2_Se-based FETs. The above results demonstrate the relatively high electronic performance of as-grown Bi_2_O_2_Se-based devices.

**Figure 4:**
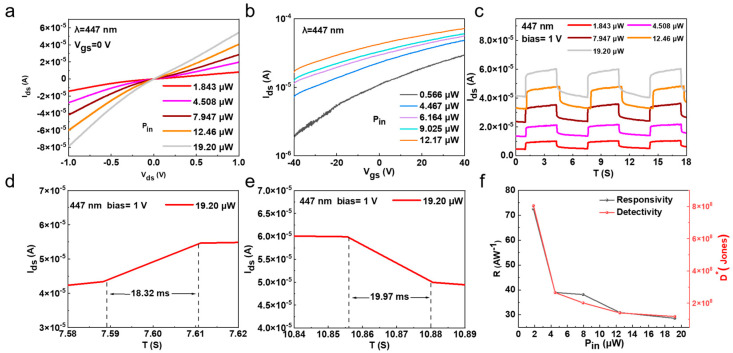
Optoelectronic performance of 2D Bi_2_O_2_Se photodetectors. (**a**) Output characteristics (Ids-Vds) of zero-gate Bi_2_O_2_Se photodetector under 447 nm laser illumination at different intensities. (**b**) Transfer characteristics (Ids-Vgs) of device under 447 nm laser illumination at different intensities with bias voltage of 1 V. (**c**) On/off switching performance of photocurrent under bias of 1 V at different laser powers. (**d**,**e**) Time-resolved photocurrent response of photodetector, showing rise and fall times of 18.32 ms and 19.97 ms, respectively. (**f**) Photoresponsivity and detectivity of Bi_2_O_2_Se photodetector versus various incident laser powers.

The above Bi_2_O_2_Se-based FET with its high mobility and on/off ratio demonstrates its potential in optoelectronic devices. Hence, we systematically studied its optoelectronic behaviors by using different incident light wavelengths and powers, as well as applying different drain and gate voltages. Figure 4a shows the output curves (Ids-Vds) with 447 nm laser excitation at different light powers, which was tested at Vgs = 0 V to elucidate the intrinsic behavior of the Bi_2_O_2_Se photodetector. The device reached its maximum photocurrent of 5.45 × 10^−5^ A at a bias voltage of 1 V and an incident laser power of 19.20 µW. Figure 4b shows the Ids-Vgs curves at a bias voltage of 1 V with different laser powers. The gate voltages range from −40 V to 40 V. Figure S5 (Supporting Information) presents the results of the *I_ph_*-*P* relationship curve. It can be observed that *I_ph_* increases with an increasing laser power as the enhancement in incident light power leads to a higher carrier concentration in the material, resulting in a stronger photocurrent. The relationship between *I_ph_* and *P* can be fitted using the following power-law equation [39]:

*I_ph_*~*P^α^* (3)

where α is the power-law exponent, obtained from data fitting as 0.736.

As the laser intensity increases, the photocurrents of both the on state and off state increase a lot, which may be due to the photogenerated electrons in the Bi_2_O_2_Se channel. Since the response time is a key parameter for photodetectors, we measured the time-resolved photo response of the photodetectors by periodically turning the laser on and off under different laser powers ranging from 1.843 µW to 19.20 µW. As shown in Figure 4c, the photodetector exhibited good on/off characteristics with excellent repeatability. Additionally, the photoinduced polarization enhancement in Bi_2_O_2_Se modulates the Schottky barrier and reduces resistance, causing the dark current to increase with increasing light power. Meanwhile, photogenerated carriers fill trap states, with some carriers remaining trapped, making it difficult for the dark current to recover. Furthermore, the magnified response curves shown in Figure 4d,e were used to calculate the rise and decay times. The rise time is defined as the time taken for the photocurrent to increase from 10% to 90% of Ids (max), and the decay time is the reverse, resulting in a rise time and a decay time of 18.32 ms and 19.97 ms, respectively, for the Bi_2_O_2_Se photodetector. For photodetectors, responsivity (*R*) and detectivity (D*) are important parameters for evaluating performance. Hence, we calculated the *R* and D* values of the photodetector versus incident laser powers, as shown in Figure 4f. The *R* value, external quantum efficiency (EQE), noise equivalent power (NEP), and detectivity (D*) were determined using the following equations [40]:

(4) $$R=\frac{I_{ph}}{P_{in}\times A_{0}}$$

$A_{0}$ and $P_{in}$ represent the active area of the device and the incident light power density, respectively. The external quantum efficiency (*EQE*) is determined using the following equation:

(5) $$EQE=\frac{h\times c\times R}{e\times \lambda }$$

where h, e, c, and λ represent Planck’s constant, elementary charge, the speed of light, and the wavelength of the illumination source, respectively. The noise equivalent power (*NEP*) is the minimum detectable power density of the photodetector (*P_in_*) with a 1 Hz bandwidth and can be defined as follows:

(6) $$NEP=\frac{\sqrt{2e\times I_{d}}}{R}$$

D* is determined by the following equation:

(7) $$D^{*}=\frac{R\times \sqrt{A_{0}}}{NEP}$$

At V_gs_ = 0 V, the Bi_2_O_2_Se photodetector achieved a maximum *R* of 72.52 AW^−1^ and a maximum D* of 8.04 × 10^8^ Jones. The R value decreases with increasing incident light power (*P_in_*), primarily influenced by changes in photoconductive gain (*G*) [41,42]. The GG is given by the following equation:

(8) $$G=(\frac{T_{t}}{T_{r}})(1+\frac{\mu_{h}}{\mu_{e}})$$

where $\mu_{h}$ and $\mu_{e}$ represent the carrier mobility of holes and electrons, respectively, and $T_{t}$ and $T_{r}$ correspond to the hole transit time and recombination lifetime. A higher *h*^+^ trap concentration suppresses *e*^−^-*h*^+^ recombination, resulting in a recombination time that is much longer than the transit time, leading to an increased *G* value and, consequently, a higher *R* value at low *P_in_*. Additionally, the detectivity (D*) follows the same trend as *R* [43]. At a wavelength of 447 nm, the *EQE* and *NEP* values were calculated, as shown in Figure S6 (Supporting Information). The *NEP* increases with incident light power, whereas the *EQE* exhibits the opposite trend. The calculated minimum *NEP* and maximum *EQE* values are 1.84 × 10^−14^ WHz^−1/2^ and 2011.89%, respectively.

Additionally, we tested the optoelectronic performance of the Bi_2_O_2_Se photodetector at 520 nm and 637 nm wavelengths. As shown in Figure S7a–d (Supporting Information), the maximum R of the Bi_2_O_2_Se photodetector reached 94.98 AW^−1^, and the maximum D* was 1.46 × 10^9^ Jones under a 520 nm laser. Figure S8a–d (Supporting Information) show that the maximum *R* and D* values of the Bi_2_O_2_Se photodetector are 64 AW^−1^ and 1.5 × 10^9^ Jones, respectively, with a 637 nm laser. Taken together, the Bi_2_O_2_Se photodetector achieved the highest *R* and D* values with 520 nm laser illumination. As seen in the comparision in Table S1 (Supporting Information), Bi_2_O_2_Se exhibits a higher responsivity and considerable detectivity compared to other photodetector materials.

## 2. Experimental Section

### 2.1. CVD Growth of Bi_2_O_2_Se

Two-dimensional Bi_2_O_2_Se flakes were grown using the CVD method. High-purity bismuth selenide powder (Bi_2_Se_3_, 48 mg) and bismuth oxide powder (Bi_2_O_3_, 12 mg) were placed in separate boats inside the tube furnace, with Bi_2_Se_3_ positioned at the center and Bi_2_O_3_ placed 4 cm away from the center. Mica substrates (1 × 2 cm^2^) were used as the growth substrate and positioned 12–16 cm downstream from the center of the furnace. The center of the furnace was heated to 820 °C with a heating rate of 40 °C/min, and this temperature was maintained for 1 h to conduct the growth. Additionally., 40 sccm Ar gas was used as carrier gas to transport reaction vapors. After the growth, the furnace was naturally cooled to room temperature in the presence of Ar.

### 2.2. Density Functional Theory Calculation

The first-principles calculations were implemented in the Vienna Ab initio Simulation Package (VASP). The generalized gradient approximation (GGA) with Perdew–Burke–Ernzerhof (PBE) were adopted for exchange and correlation interactions. Bulk Bi_2_O_2_Se was chosen as crystal structure. For the calculation, the Hellman–Feynman forces were set to less than 0.02 eV Å, and the energy cutoff was set to 400 eV. The K points were sampled by Monkhorst-Pack with a 7 × 7 × 7 mesh. The single electronic step was converged to 1 × 10^−9^ eV.

### 2.3. Device Fabrication

Bi_2_O_2_Se flakes on a mica substrate were transferred onto a Si/SiO_2_ substrate using a 1.5% polystyrene (PS) solution. The electrodes were patterned via photolithography. The pattern was developed using a 25% aqueous solution of tetrame-thylammonium hydroxide, which was diluted with deionized water at a ratio of 3:25. Subsequently, a 5 nm Cr layer and a 50 nm Au layer were deposited via thermal evaporation.

### 2.4. Characterization

The sizes and shapes of Bi_2_O_2_Se flakes were characterized by using optical microscopy (Sunny Optical CX40M, Yuyao, China). The thickness of Bi_2_O_2_Se flakes was measured by AFM (Cypher ES, Asylum Research, Santa Barbara, CA, USA). Raman spectra were collected with a confocal microscope spectrometer (Renishaw LabRAM Invia, Wotton-under-Edge, UK). The element chemical states of Bi_2_O_2_Se flakes were tested using XPS (Thermo Scientific Escalab 250Xi, Waltham, MA, USA) with Al-ka (1486.6 eV) as a source. The electronic performance of FET and photodetectors was tested using a probe station and a semiconductor analyzer (Keithley 2634B, Cleveland, OH, USA), with the laser power calibrated using a power meter.

## 3. Conclusions

In summary, we developed an atmospheric pressure CVD method for the growth of 2D thin-layer Bi_2_O_2_Se. By adjusting the growth parameters, we achieved precise control over the sizes and shapes of Bi_2_O_2_Se flakes. The thinnest Bi_2_O_2_Se flake obtained was 4 nm, and the largest Bi_2_O_2_Se flake was 220 µm. The role of vapor flux of precursors was studied to bridge the growth parameters and vapor concentration. The FETs fabricated from Bi_2_O_2_Se flakes exhibited an n-type transport behavior, with an electron mobility of 129 cm^2^V^−1^s^−1^ and an on/off ratio of 4.51 × 10^5^. Additionally, the corresponding photodetectors showed a responsivity of 94.98 AW^−1^ and a detectivity of 1.46 × 10^9^ Jones. Our study demonstrates that 2D Bi_2_O_2_Se flakes are promising for high-performance optoelectronics.

## Data Availability

Data are contained within the article and Supplementary Materials.

## Supplementary Materials

The following supporting information can be downloaded at https://www.mdpi.com/article/10.3390/nano15080567/s1. Figure S1. Optical microscope image of large domain-sized Bi_2_O_2_Se flakes grown on a mica substrate. Figure S2. The Raman spectra of Bi_2_O_2_Se flakes with different thicknesses under 532 nm laser excitation. (a,b) The insets show the optical microscope images of the Bi_2_O_2_Se flakes, with the main graphs displaying the Raman spectra. The A_1g_ peaks are approximately 158.14 cm^−1^ in (a) and 160.00 cm^−1^ in (b). Figure S3. Atomic force microscope images and height profiles of different Bi_2_O_2_Se samples, with thicknesses of (a) 5.2 nm, (b) 4 nm, and (c) 7.8 nm. Figure S4. Effect of temperature on the lateral sizes of Bi_2_O_2_Se flakes. (a–e) Representative optical microscope images of Bi_2_O_2_Se flakes grown at different temperatures (800 °C, 810 °C, 820 °C, 830 °C, 840 °C), and (f–j) the statistics of corresponding lateral size distribution. Scale bar: 50 µm. Figure S5. The Iph-P relationship curve under 447 nm illumination, measured at room temperature. Figure S6. Quality curves of NEP and EQE for the Bi_2_O_2_Se photodetector under 447 nm illumination at different power levels. Figure S7. The performance of 2D Bi_2_O_2_Se photodetectors with 520 nm laser. (a) Output characteristic curves (I_ds_-V_ds_) of the zero-gate Bi_2_O_2_Se photodetector under different laser power. (b) Transfer characteristic curves (I_ds_-V_gs_) of the device at V_ds_ = 1 V under different laser power. (c) On/off testing of the photocurrent under 1V bias with different laser power levels. (d) The responsivity and detectivity of the photodetector under different laser power. Figure S8. The performance of 2D Bi_2_O_2_Se photodetectors with 637 nm laser. (a) Output characteristic curves (I_ds_-V_ds_) of the zero-gate Bi_2_O_2_Se photodetector under different laser power. (b) Transfer characteristic curves (I_ds_-V_gs_) of the device at V_ds_ = 1 V under different laser power. (c) On/off testing of the photocurrent under 1 V bias with different laser power levels. (d) The responsivity and detectivity of the photodetector under different laser power. Table S1. Performance comparison of various photodetectors based on common two-dimensional materials. Refs. [44,45,46,47,48,49,50,51,52,53] are cited in the supplementary materials.
