# Supplementary material for: Revealing the Role of Vapor Flux in Chemical Vapor Deposition Growth of Bi2O2Se for Photodetectors
Source: Nanomaterials (Basel). 2025 Apr 8;15(8):567. doi: 10.3390/nano15080567 (PMC12029218; doi:10.3390/nano15080567)
Supplement: Supplementary file 1 [file nanomaterials-15-00567-s001.zip › nanomaterials-3538567-supplementary.pdf]

# Revealing the Role of Vapor Flux in Chemical Vapor Deposition Growth of $Bi_2O_2Se$ for Photodetectors

Qin Huang, Jiqing Nie, Jian Li, Meng Wang, Changyuan Ding, Haiyan Nan, Xiaofeng Gu and Zhengyang Cai *

School of Integrated Circuits, Jiangnan University, Wuxi 214122, China

*Corresponding Author: caizy@jiangnan.edu.cn

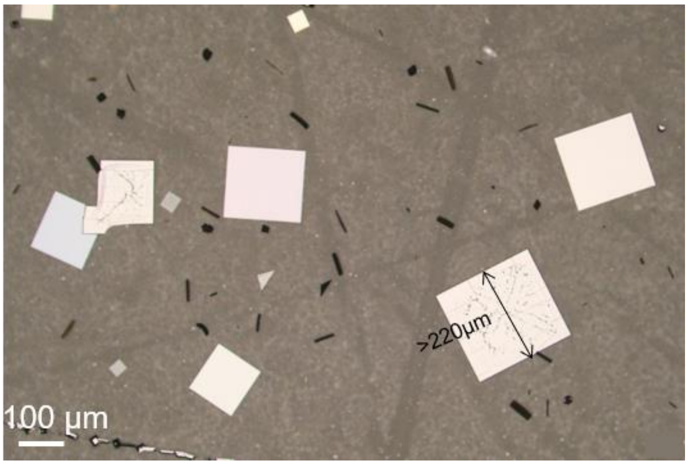


**Figure S1**. Optical microscope image of large domain-sized $Bi_2O_2Se$ flakes grown on a mica substrate.

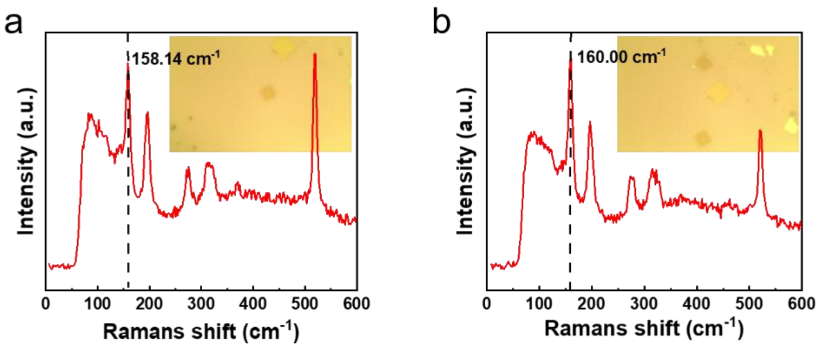


**Figure S2**. The Raman spectra of $Bi_2O_2Se$ flakes with different thicknesses under 532 nm laser excitation. (a-b) The insets show the optical microscope images of the $Bi_2O_2Se$ flakes, with the main graphs displaying the Raman spectra. The $A_{1g}$ peaks are approximately 158.14 $cm^{-1}$ in (a) and 160.00 $cm^{-1}$ in (b).

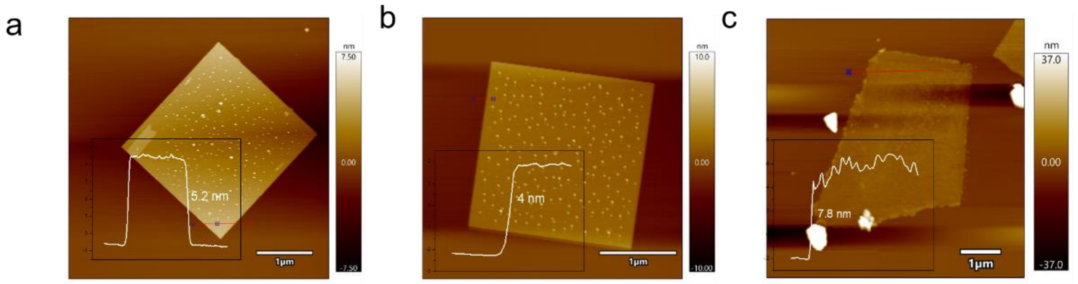


**Figure S3**. Atomic force microscope images and height profiles of different $Bi_2O_2Se$ samples, with thicknesses of (a) 5.2 nm, (b) 4 nm, and (c) 7.8 nm.

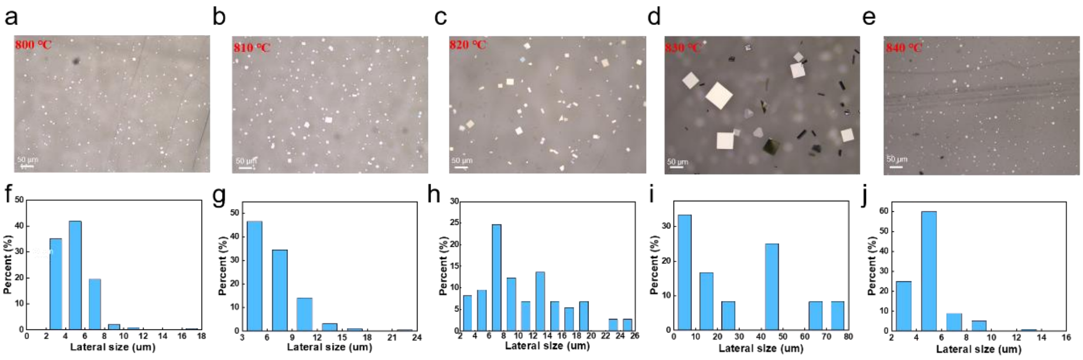


**Figure S4**. Effect of temperature on the lateral sizes of $Bi_2O_2Se$ flakes. (a-e) Representative optical microscope images of $Bi_2O_2Se$ flakes grown at different temperatures (800 °C, 810 °C, 820 °C, 830 °C, 840 °C), and (f-j) the statistics of corresponding lateral size distribution. Scale bar: 50 μm.

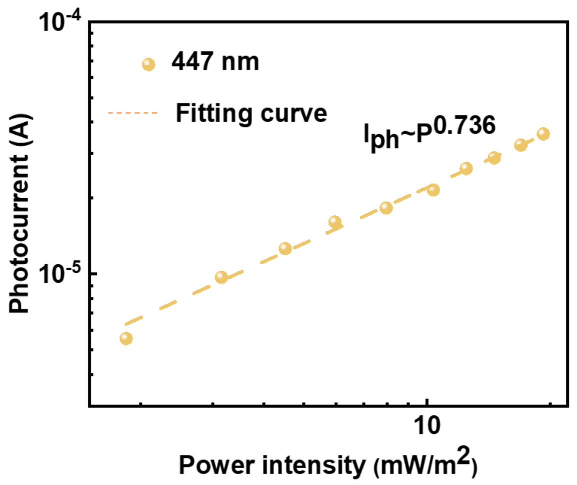


**Figure S5**. The Iph-P relationship curve under 447 nm illumination, measured at room temperature.

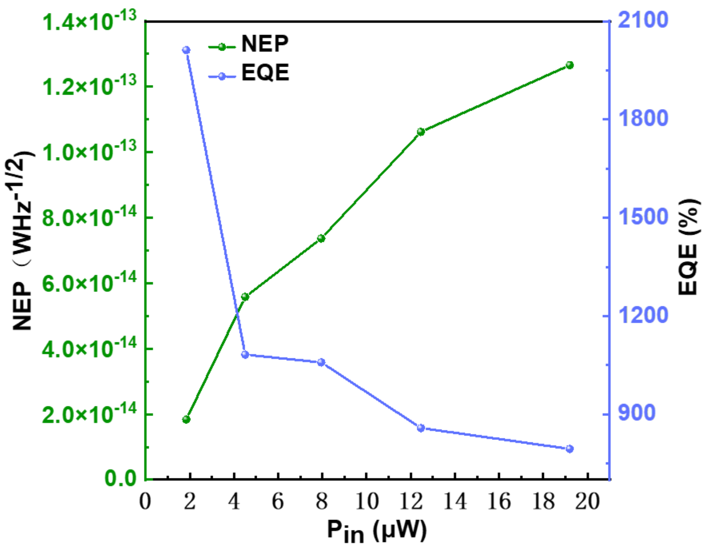


**Figure S6.** Quality curves of NEP and EQE for the $Bi_2O_2Se$ photodetector under 447 nm illumination at different power levels.

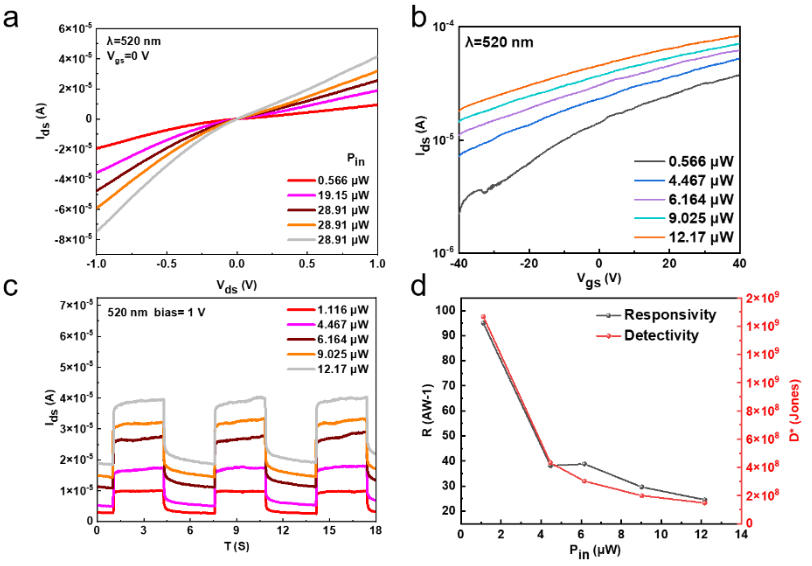


**Figure S7**. The performance of 2D $Bi_2O_2Se$ photodetectors with 520 nm laser. (a) Output characteristic curves ($I_{ds}$-$V_{ds}$) of the zero-gate $Bi_2O_2Se$ photodetector under different laser power. (b) Transfer characteristic curves ($I_{ds}$-$V_{gs}$) of the device at $V_{ds}$=1 V under different laser power. (c) On/off testing of the photocurrent under 1V bias with different laser power levels. (d) The responsivity and detectivity of the photodetector under different laser power.

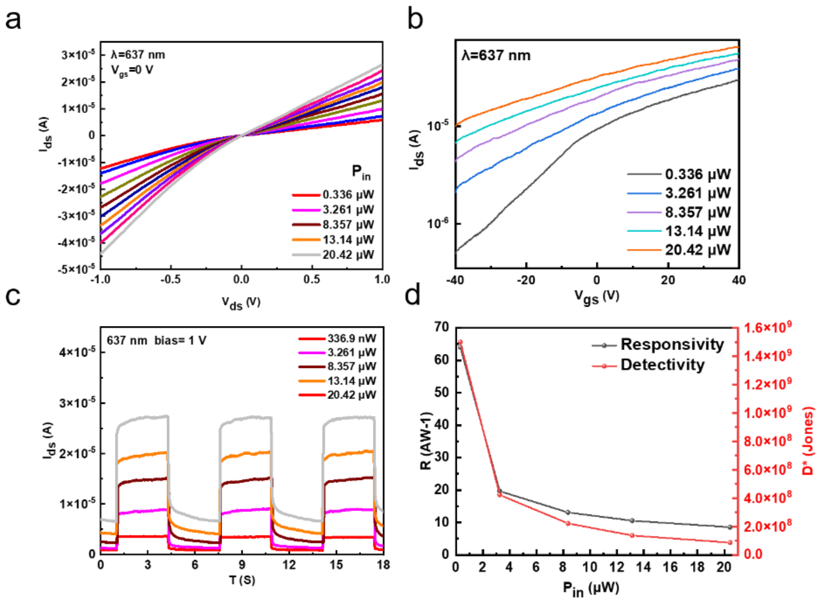


**Figure S8**. The performance of 2D $Bi_2O_2Se$ photodetectors with 637 nm laser. (a) Output characteristic curves ($I_{ds}$-$V_{ds}$) of the zero-gate $Bi_2O_2Se$ photodetector under different laser power. (b) Transfer characteristic curves ($I_{ds}$-$V_{gs}$) of the device at $V_{ds}$=1 V under different laser power. (c) On/off testing of the photocurrent under 1V bias with different laser power levels. (d) The responsivity and detectivity of the photodetector under different laser power.

**Table 1.** Performance comparison of various photodetectors based on common two-dimensional materials.

| Material | Responsivity(A $W^{-1}$) | $D^*$(Jones) | Ref. |
|---|---|---|---|
| $Bi_2O_2Se$ | 94.98 | $1.46\times10^9$ | Our work |
| Graphene | 33.8 | | [1] |
| $MoS_{2x}Se_{2(1-x)}$ | 2.4 x $10^5$ | | [2] |
| BP | 6 | | [3] |
| Te | 383 | | [4] |
| $SnS_2$ | 91.7 | | [5] |
| SnSe | 0.16 | $3.9x10^7$ | [6] |
| SnTe | 0.698 | $3.9x10^9$ | [7] |
| SnS | 920 | $1.09x10^9$ | [8] |
| InSe | 19300 | 3 $x10^{13}$ | [9] |
| BiOBr | 12739.13 | 8.37 $x10^{12}$ | [10] |

## References


1. Fukushima, S.; Shimatani, M.; Okuda, S.; et al. High responsivity middle-wavelength infrared graphene photodetectors using photo-gating. *Appl. Phys. Lett.* **2018**, *113(6).*

2. Yin, L.; He ,P.; Cheng, R.; et al. Robust trap effect in transition metal dichalcogenides for advanced multifunctional devices. *Nat. Commun.* **2019**, *10(1),* 4133.

3.Wang, J.; Rousseau, A.; Eizner, E, et al. Spectral responsivity and photoconductive gain in thin film black phosphorus photodetectors. *ACS Photonics,* **2019**, *6(12),* 3092-3099.

4.Shen, C.; Liu, Y.; Wu, J.; et al. Tellurene photodetector with high gain and wide bandwidth. *ACS Nano,* **2019**, *14(1),* 303-310.

5.Lei, Y.; Luo, J.; Yang, X.; et al. Thermal evaporation of large-area $SnS_2$ thin films with a UV-to-NIR photoelectric response for flexible photodetector applications. *ACS Appl. Mater. Interfaces,* **2020**, *12(22),* 24940-24950.

6.Xu, H.; Hao, L.; Liu, H.; et al. Flexible SnSe photodetectors with ultrabroad spectral response up to 10.6 μm enabled by photobolometric effect. *ACS Appl. Mater. Interfaces,* **2020**, *12(31),* 35250-35258.

7.Liu, J.; Li, X.; Wang, H.; et al. Ultrathin high-quality SnTe nanoplates for fabricating flexible near-infrared photodetectors. *ACS Appl. Mater. Interfaces,* **2020,** *12(28),* 31810-31822.

8.Krishnamurthi, V.; Khan, H.; Ahmed, T.; et al. Liquid-metal synthesized ultrathin SnS layers for high-performance broadband photodetectors. *Adv. Mater.* **2020**, *32(45),* 2004247.

9.Jang, H.; Seok, Y.; Choi, Y. T.; et al. High-performance near-infrared photodetectors based on surface-doped InSe. *Adv. Funct. Mater.* **2021**, *31(3)*, 2006788.

10.Gong, C.; Chu, J.; Qian, S.; et al. Large-scale ultrathin 2D wide-bandgap BiOBr nanoflakes for gate-controlled deep-ultraviolet phototransistors. *Adv. Mater.* **2020**, *32(12)*, 1908242.
